# CX3CR1 at V249M and T280M Gene Polymorphism and Its Potential Risk for End-Stage Renal Diseases in Egyptian Patients

**DOI:** 10.1155/2021/6634365

**Published:** 2021-04-24

**Authors:** Asmaa Fathelbab Ibrahim, Asmaa Osama Bakr Seddik Osman, Lamiaa M. Elabbasy, Mostafa Abdelsalam, A. M. Wahab, Maysaa El Sayed Zaki, Radwa Ahmed Rabea Abdel-Latif

**Affiliations:** ^1^Lecturer of Clinical & Chemical Pathology, Faculty of Medicine, Beni Suef University, Beni Suef, Egypt; ^2^Clinical Pathology Department, Faculty of Medicine, Assiut University, Asyut, Egypt; ^3^Department of Medical Biochemistry, Faculty of Medicine, Mansoura University, Mansoura, Egypt; ^4^Department of Biochemistry, College of Medicine, Almaarefa University, Riyadh, Saudi Arabia; ^5^Mansoura Nephrology and Dialysis Unit, Internal Medicine Department, Faculty of Medicine, Mansoura University, Mansoura, Egypt; ^6^Clinical Pathology Department, Faculty of Medicine, Mansoura University, Mansoura, Egypt

## Abstract

CX3CL1-CX3CR1 pathway may be one of the future treatment targets to delay the progression of end-stage renal diseases. This study aimed to evaluate the CX3CR gene polymorphism in Egyptian patients with ESRD and its relation to fractalkine blood level. The study included 100 patients with ESRD on dialysis, 61 males and 39 females with mean age 51.02 ± 7.8 years. The V2491 genotype revealed a significant increase in the frequency of GG genotype in healthy control (83%) compared to patients [69%] with a significant increase in GA in patients [30%] compared to control subjects [15%], *P* = 0.03. T280M study showed a statistically significant prevalence of TT genotype in healthy control subjects [86%-OR 95% CI 1.7] compared to patients [70%] with a significant increase in the prevalence of TA in patients [29%] compared to control subjects [13%], *P* = 0.01. There was a significant increase in fractalkine levels in genotypes GA + AA [503.04±224.1] pg/ml compared to genotype GG [423.6 210.3], *P* = 0.03. Moreover, there was a significant increase in the blood level of fractalkine in genotype TA + AA [498.8 219.6] compared to genotype TT [426.8±212.8], *P* = 0.05. In conclusion, our study showed that both V2491-GA genotype and T280M-TA are associated with potential risk for end-stage renal disease in Egyptian patients.

## 1. Introduction

Chronic kidney disease is a major health concern worldwide. This disease leads to reduced quality of life with high rates of mortality. There is a need for early diagnosis and monitoring of the progression of the disease for good management. The definition of chronic renal diseases involves proteinuria and reduced glomerular filtration rate. The definition includes reduced glomerular filtration rate [GFR] less than 60 mL/min and the presence of albumin more than 30 mg per gram of creatinine with the presence of abnormalities of kidney structure or function more than three months. Moreover, end-stage renal disease is defined as a GFR of less than 15 mL/min [1, 2].

Chemokine fractalkine [CX3CL1] is a transmembrane molecule expressed in two forms; one form expressed on the endothelium cells, and the other form is present as a soluble form in the blood. Chemokine fractalkine molecule is activated by proinflammatory cytokines to promote T lymphocytes and monocytes' retention, while the soluble form induces chemotaxis [3].

Receptor 1 for CX3C chemokine [CX3CR1], a 7-transmembrane domain G-protein-coupled receptor, is expressed by natural killer cells, monocytes, CD8 T, and CD4 T-cells [4, 5]. There is accumulating evidence that the CX3CL1-CX3CR1 pathway is responsible for the pathogenesis of various diseases such as atherosclerosis, chronic kidney disease, and solid cancer [5–7]. The expression of fractalkine and its receptor on cells has been shown in renal abnormalities [8–12]. The fractalkine system may promote renal inflammation and renal fibrogenesis [13]. The use of anti-CX3CR1 antibody treatment to the glomeruli in experimental study leads to the improvement of renal function [14] and protects from acute renal ischemia [15].

The single nucleotide polymorphisms [SNPs] in the gene coding for fractalkine receptors are associated with changes in the receptor's amino acids' conserved region. These SNPs were related to various diseases such as atherosclerosis and ESRD. The common two SNPs are V249I and T280, and the detection of these SNPs may have a role in early diagnosis of ESRD with the possibility of the prevention of the disease progression by using anti-CX3CR1 therapy [14, 16–18].

There were limited studies about the association of CX3CR single nucleotide polymorphisms and the presence of end-stage renal diseases.

The present study aimed to evaluate the presence of SNPs of CX3CR at V249I and T280 in association with end-stage renal disease and its relation to fractalkine blood level.

## 2. Material and Method

The study was a case-control study. The study included 100 patients with end-stage renal disease recruited from Mansoura Nephrology and Dialysis Unit, Mansoura University, from January 2018 till March 2019. The patients were adults with a glomerular filtration rate below 30 ml/minute with no history of infection or malignancy within three months before the study's recruitment. In addition, 100 healthy subjects were included as a control subject. The Mansoura Ethical committee approved the study and provided written consent was obtained from each participant.

Each participant was subjected to full clinical history taking and clinical examination. Ten melilite blood samples were obtained from each subject for laboratory investigation.

The blood sample was subjected to laboratory measurement of creatinine, urea, total cholesterol, HDL, total triglyceride, and LDL-cholesterol determination by spinreact kits [SPINREACT Crta. Sta. Coloma, · 17176 St. Esteve d'en Bas · Girona-Spain]. A complete blood count was performed by sysmex. High sensitive C-reactive protein was measured by enzyme-linked immunosorbent assay [ELISA] by kit [Eagle Biosciences-10 Columbia Dr, Amherst, NH 03031-USA]. The determination of fractalkine was determined by ELISA kit [Human CX3CL1/Fractalkine Quantikine-614 McKinley Place NE, Minneapolis, MN 55413, USA].

### 2.1. Fractalkine Quantikine Principle of the Assay

This assay employs the quantitative sandwich enzyme immunoassay technique. A monoclonal antibody specific for human fractalkine has been precoated onto a microplate. Standards and samples are pipetted into the wells, and the immobilized antibody bounds any fractalkine present. After washing away any unbound substances, an enzyme-linked monoclonal antibody specific for human fractalkine is added to the wells. Following a wash to remove any unbound antibody-enzyme reagent, a substrate solution is added to the wells, and color develops in proportion to the amount of fractalkine bound in the initial step. The color development is stopped, and the intensity of the color is measured.

### 2.2. Polymerase Chain Reaction (PCR)-Restriction Fragment Length Polymorphism (RFLP) for CX3CR at V249I and T280

#### 2.2.1. Extraction of DNA

DNA was extracted from the blood samples by using the Qiagen extraction kit from whole blood by using QIAamp DNA Blood Mini Kit [Qiagen-Germany] according to the manufacturer protocol. The extracted DNA was kept at −80°C till the time of the amplification.

#### 2.2.2. PCR-RFLP for CX3CR1 Gene T280M and V249I

The first step was PCR for amplification of CX3CR1 gene T280M and V249I by the use of the primers with sequences F: 5′-CCGAGGTCCTTCAGGAAATCT-3′] and R: 5′-TCAGCATCAGGTTCAGGAACTC-3′. The amplification process was performed by the use of Qiagen ready to use amplification kit [Qiagen-Germany]. The amplification reaction was performed by the thermal cycler. The amplification process included the following steps: 1 minute of denaturation at 94°C, followed by 34 cycles of 30 seconds' denaturation at 94°C, 40 seconds' annealing at 50°C, and 55 seconds' extension at 72°C. The amplified products' bp 588 was subjected to the restriction by the enzymes BsmBI and Psp1406I for T280M and V249I polymorphisms, respectively.

The PCR products were digested for 2 hours at 55°C with *Bsm*BI and 37°C with Psp1406I and checked on 2.5% agarose gels. Two restriction sites for BsmBI are present at positions 216 and 291 of the normal strand [T280], which was completely digested into three fragments of 75, 216, and 297 bp; the second site is disrupted in the mutated strand [M280], thus displaying only two fragments of 216 and 372 bp. Four bands [75, 216, 297, and 372 bp] were present in heterozygous subjects. The V249I polymorphism was detected using Psp1406I. One restriction site for Psp1406I is present at position 205 of the normal strand, splits into two fragments of 205 and 383 bp. This site was disrupted in the mutated strand, which remained undigested [588 bp] [18].

### 2.3. Statistical Analysis

Results were analyzed using SPSS 24. One-way ANOVA and *T*-tests analyzed continuously variable data. Categorical data were compared by *χ*^2^ tests. Odds ratios [95% confidence interval] were calculated to detect risk ratio. *P* was considered significant if it was <0.05.

## 3. Results

The included 100 patients with end-stage renal failure on dialysis, diabetes mellitus, and hypertension were the main causes of end-stage renal disease (supplementary)(available here), 61 males and 39 females with mean age ± SD 51.02 ± 7.8 years. There was a statistically insignificant difference between age and sex distribution between patients and the control group [*P* = 0.4, *P* = 0.1, respectively]. There was a statistically significant reduction of hemoglobin level, platelets, and total calcium between patients and control subjects [*P* = 0.002, *P* = 0.004, *P* = 0.0001, respectively], while there was a significant elevation of creatinine, urea, hsCRP, and fractalkine levels in patients compared to control subjects [*P* = 0.0001], Table 1.

The study of the V2491 genotype revealed a significant increase in the frequency of GG genotype in healthy control (83%) compared to patients [69%] with a significant increase in GA in patients [30%] compared to control subjects [15%], *P* = 0.03. The frequency of haplotype G had significantly increased prevalence in healthy control [90.5%] compared to control subjects [84%], with a significant increase in A haplotype in patients [16%] compared to control [9.5%], *P* = 0.05. In the study of T280M, there was a statistically significant prevalence of TT genotype in healthy control subjects [86%-OR 95% CI 1.7] compared to patients [70%] with a significant increase in the prevalence of TA in patients [29%] compared to control subjects [13%], *P* = 0.01. There was a significant increase in the prevalence of haplotype T in control subjects [92.5%] compared to patients [84.5%] with a significant increase in A haplotype in patients [15.5%] compared to control subjects [7.5%], *P* = 0.01 (Table 2).

Comparing blood levels of fractalkine between V2491 genotypes GG and GA + AA, there was a significant increase in fractalkine levels in genotypes GA + AA [503.04±224.1] pg/ml compared to genotype GG [423.6±210.3], *P* = 0.03. Moreover, there was a significant increase in the blood level of fractalkine in genotype TA + AA [498.8±219.6] compared to genotype TT [426.8±212.8], *P* = 0.05, Table 3.

Comparing demographic and laboratory data between patients with different genotypes revealed insignificant difference, Table 4.

## 4. Discussion

The fractalkine receptor chemokine receptor 1 [CX3CR1] and fractalkine ligand mediate chemotaxis and adhesion of immune cells, and both are associated with the pathogenesis and progression of various inflammatory disorders and malignancies. This axis has been linked to renal diseases as its expression leads to migration and colonization of inflammatory cells in renal tissue [19]. There is a link between the relationship between the CX3CL1/CX3CR1 axis and renal diseases and disorders, including diabetic nephropathy, renal allograft rejection, infectious renal diseases, IgA nephropathy, fibrotic kidney disease, lupus nephritis and glomerulonephritis, acute kidney injury, and renal carcinoma. On the other hand, the CX3CL1/CX3CR1 axis may play a protective role against some kidney disorders [19].

The present study's objective was to investigate the prevalence of V2491 and T280M genotypes among Egyptian patients with end-stage renal diseases and healthy control. The genotypes GG of V2491 and genotype TT of T280M were more prevalent among healthy control compared to patients. Borkar et al. reported a similar finding for V2491 [14]. On the other hand, this finding contradicts previous findings by Yadav and his colleagues [20], who stated that there was no significant difference between patients and control subjects regarding the GG genotype of V2491 and TT genotype of T280 in the Indian population.

There were previous reports that linked the association of polymorphisms in the CX3CR1 and coronary artery disease [18, 21], risk of acute rejection in renal transplant recipients [22], cancer rates in renal transplant recipient [23], age-related macular degeneration [24], and HIV [25]. The presence of V249 and T280 SNPs in the CX3CR1 gene was reported to be associated with overexpression by activated inflammatory cells and with an increase of the binding affinity for fractalkine [25, 26]. Moreover, it was reported that CX3CR1 acted in vitro as an HIV co-receptor in CD4, the M280 allele was accompanying with receptorexpression decrease on PBMCs from HIV-positive subjects, and this was associated with fast disease progression [25]. In previous studies, the overexpression of CX3CL1 and CX3CR1+ cells was related to renal disease with protective effects of macrophage depletion blocking CX3CR1 in an experimental study [27]. Therefore, the use of antibodies to CTXCR1 may have a role in preventing end-stage renal failure if used in susceptible patients with renal disease and unfavorable genotypes.

In the present study, the fractalkine level in studied subjects was significantly increased in subjects with genotype GA + AA of V2491 and TA + AA of genotypes T280M. However, the levels of fractalkine had a nonsignificant difference between patients with different genotypes. There was a limited dataabout the association between fractalkine level and genetic variants of its receptor CX3CR1 and a single study in end-stage renal disease revealed no statistically significant difference [20]. The significant increase of fractalkine level in CKD patients may reflect its role in predisposition to renal failure. Fractalkine/CX3CL1 and its receptor CX3CR are upregulated during renal injury in several mouse models, including experimental folic acid nephropathy, crescentic GN, and diabetic nephropathy [9, 28, 29]. Treatment with a neutralizing antibody against fractalkine/CX3CL1 improves renal damage by blocking crescentic formation and macrophage infiltration in rat nephrotoxic nephritis [19]. All these studies imply there might be an essential role for CCL and CX3CL chemokines in chronic renal disease regardless of inciting events.

There was an insignificant association between different genotypes in patients and their laboratory findings, including hsCRP. This finding is similar to a previous study by Yaldf et al. [20].

The study needs to be applied to large groups of Egyptian patients with longitudinal follow-up of patients with various renal impairment stages.

The present study highlights that SNP of CX3CR1 at V249M and T280M had a potential risk for end-stage renal diseases. The influence may be related to increasing section fractalkine. This pathway may be targeted as a new pathway for the therapeutic management of chronic renal disorders. Further extended longitudinal studies are needed to validate these findings.

## Figures and Tables

**Table 1:** Comparison of demographic and laboratory data between patients and control group.

	ESRD (*n* = 100)	Healthy control (*n* = 100)	*P*
Age (mean ± SD)	51.02 ± 7.8	50.1 ± 6.6	*P* = 0.4
Gender Male *n* (%) Female *n* (%)	61 [61%] 39 [39%]	49 [49%] 51 [51%]	*P* = 0.1
Hemoglobin gm/dl (mean ± SD)	10.8 ± 1.4	13.2 ± 1.7	*P* = 0.002
Total leucocytes × 10^3^/cmm (mean ± SD)	7.4 ± 5.3	7.6 ± 2.3	*P* = 0.7
Platelets × 10^3^/cmm (mean ± SD)	185.5 ± 78.9	216.1 ± 69.4	*P* = 0.004
Creatinine mg/dl (mean ± SD)	10.4 ± 1.04	1.0 ± 0.25	*P* = 0.0001
Urea mg/dl (mean ± SD)	121.5 ± 112.2	27.01 ± 2.6	*P* = 0.0001
Calcium mg/l (mean ± SD)	8.2 ± 0.7	9.6 ± 0.5	*P* = 0.0001
Cholesterol mg/dl	211.9 ± 51.5	207.8 ± 60.2	*P* = 0.6
LDL-cholesterolmg/dl (mean ± SD)	132.2 ± 47.1	125.3 ± 11.4	*P* = 0.2
Triglycerides mg/dl (mean ± SD)	129.8 ± 46.2	127.8 ± 59.1	*P* = 0.8
HDL mg/dl (mean ± SD)	45.6 ± 13.04	45.6 14.04	*P* = 0.7
Fractalkine pg/ml (mean ± SD)	631.03 ± 144.2	254.3 ± 33.7	*P* = 0.0001
hsCRP mg/l (mean ± SD)	114.3 ± 31.3	71.9 ± 16.2	*P* = 0.0001

Data were expressed as mean ± SD. SD: standard deviation.

**Table 2:** Comparison of fractalkine V2491 and T280M genotypes between patients and control.

	ESRD (*n* = 100)	Healthy control (*n* = 100)	OR (95%)	*P*
V2491				
GG *n* (%)	69 [69%]	83 [83%]	1.5 [1.03–2.3]	*P* = 0.03^*∗*^
GA *n* (%)	30 [30%]	15 [15%]		
AA *n* (%)	1 [1%]	2 [2%]		
G *n* (%)	168 [84%]	181 [90.5%]		*P* = 0.05^*∗∗*^
A *n* (%)	32 [16%]	19 [9.5%]		

T280M				
TT *n* (%)	70 [70%]	86 [86%]	1.7 [1.1–2.7]	*P* = 0.01^*∗*^
TA *n* (%)	29 [29%]	13 [13%]		
AA *n* (%)	1 [1%]	1 [1%]		
T *n* (%)	169 [84.5%]	185 [92.5%]		*P* = 0.01^*∗∗*^
A *n* (%)	31 [15.5%]	15 [7.5%]		

^*∗*^For genotypes. ^*∗∗*^For alleles.

**Table 3:** Correlation between genotypes of V2491 and T280M and fractalkine level.

Genotype	Fractalkine mean ± SD/pg/ml	*P*
V2491		
GG	423.6 ± 210.3	*P* = 0.03
GA + AA	503.04 ± 224.1	

T280M		
TT	426.8 ± 212.8	*P* = 0.05
TA + AA	498.8 ± 219.6	

Data were expressed as mean ± SD. SD: standard deviation.

**Table 4:** Comparison of demographic and laboratory data between patients with different genotypes of fractalkine.

	V2491			T280M		
	GG	GA + AA	*P*	TT	TA + AA	*P*
Age mean ± SD	50.4 ± 7.6	58.4 ± 7.6	0.2	50.8 ± 7.7	51.5 ± 8.2	0.7
Gender						
Male *n*(%)	41 [67.2%]	20 [22.8%]	0.4	40 [65.6%]	21 [34.4%]	0.2
Female *n*(%)	28 [71.8%]	11 [28.2%]		30 [76.9%]	9 [23.1%]	
HB mean ± SD	11.1 ± 8.9	10.2 ± 1.8	0.6	10.0 ± 1.9	10.6 ± 2.00	0.1
WBCs mean ± SD	7.7 ± 6.1	6.6 ± 2.7	0.4	7.5 ± 6.1	7.1 ± 2.8	0.7
Platelets mean ± SD	184.7 ± 80.1	186.7 ± 78.7	0.9	182.4 ± 79.1	192.1 ± 81.0	0.6
Urea mean ± SD	128.6 ± 13.1	105.8 ± 24.3	0.3	109.6 ± 24.3	149.1 ± 20.1	0.1
Creatinine mean ± SD	10.4 ± 1.0	10.3 ± 0.9	0.7	10.4 ± 1.03	10.9 ± 1.1	0.3
Cholesterol mean ± SD	217.9 ± 48.7	198.6 ± 56.7	0.1	207.1 ± 52.6	223.4 ± 47.8	0.1
LDL mean ± SD	138.4 ± 50.5	118.3 ± 35.8	0.05	131.7 ± 49.9	133.4 ± 40.7	0.8
TGs mean ± SD	130.8 ± 47.02	127.5 ± 45.1	0.7	134.4 ± 48.1	189.1 ± 40.4	0.1
HDL mean ± SD	44.8 ± 12.1	47.2 ± 17.8	0.4	44.6 ± 14.7	44.7 ± 12.2	0.3
Fra mean ± SD	628.1 ± 138.6	637.6 ± 158.3	0.76	636.9 ± 136.4	617.4 ± 158.5	0.5
hsCRP mean ± SD	90.2 ± 16.8	94.6 ± 15.6	0.22	90.9 ± 16.8	93.3 ± 15.9	0.5

Data were expressed as mean ± SD. SD: standard deviation.

## Data Availability

The data used to support the findings of this study are available from the corresponding author upon request.

## Abbreviations

ESRD: End-stage renal disease
GFR: Glomerular filtration rate
CX3CL1: Chemokine fractalkine
CX3CR1: Receptor 1 for CX3C chemokine
SNPs: Single nucleotide polymorphisms.

## Supplementary Materials

Supplementary Materials Causes of end-stage renal diseases.
