# Supplementary material for: CX3CR1 at V249M and T280M Gene Polymorphism and Its Potential Risk for End-Stage Renal Diseases in Egyptian Patients
Source: Int J Nephrol. 2021 Apr 24;2021:6634365. doi: 10.1155/2021/6634365 (PMC8093072; doi:10.1155/2021/6634365)
Supplement: Supplementary Materials — Causes of end-stage renal diseases. [file 6634365.f1.docx]

| Etiology | |
| --- | --- |
| Hypertension | 17 |
| Diabetes mellitus | 41 |
| Chronic pyelonephritis | 9 |
| Combined hypertension and diabetes | 25 |
| Lupus nephritis | 5 |
| Chronic glomerulonephritis | 3 |
